# Algal Biomass and Algal-Derived Preparations in Livestock Nutrition: Gut Microbiota Modulation, Host Molecular Pathways, and Bioprocess Innovations

**DOI:** 10.3390/vetsci13101007

**Published:** 2026-09-24

**Authors:** Fei Lu, Ziming Liu, Bing Cao, Chengyang Wu, Peidong He, Lihua Li, Zhongwei Chen, Yuehao Ge

**Affiliations:** 1 Nantong Marine Center, Ministry of Natural Resources, Nantong 226000, China; 13625222109@163.com (F.L.); semeicaobing@163.com (B.C.); w891684032@163.com (C.W.); hpdriyue@163.com (P.H.); lilihua02200327@163.com (L.L.); 2 Nantong Natural Resources Affairs Service Center, Nantong 226000, China; 19552606600@163.com

**Keywords:** algae, microalgae, cyanobacteria, macroalgae, livestock nutrition, gut microbiota, bioactive compounds, host signaling pathways

## Abstract

Algal biomass and algal-derived preparations have attracted increasing interest in livestock nutrition because of their potential to modulate the gut microbiota, which plays a crucial role in animal health, immunity, and growth performance. This review focuses primarily on microalgae and cyanobacterial preparations, while also considering selected macroalgae relevant to livestock feeding, without restricting its scope to marine sources. In this narrative review, we summarize recent advances in the physicochemical properties and biological activities of key algal components, such as polysaccharides, polyunsaturated fatty acids, and carotenoids. We assess evidence for changes in potentially beneficial bacteria, including *Lactobacillus* and *Bifidobacterium*, and for inhibitory effects against selected pathogenic bacteria, including *Salmonella* and pathogenic strains of *Escherichia coli*. We also examine proposed links between these compounds, microbial short-chain fatty acid and bile acid metabolism, and host immune and growth responses. This review discusses practical applications of algal biomass and algal-derived preparations in livestock production, with a focus on inclusion levels and safety assessments. Effects are evaluated in relation to the source organism, preparation, and animal model, rather than assumed to be shared across algal groups. Current challenges and future research directions are also identified to facilitate the efficient utilization of algal resources in sustainable animal husbandry. This review identifies the evidence supporting specific feeding applications and distinguishes observed animal responses from proposed mechanisms.

## Simple Summary

The use of antibiotics in livestock production has raised concerns regarding antimicrobial resistance and animal health, creating a need for safe and sustainable alternatives. Algae contain various natural bioactive compounds, including polysaccharides, omega-3 fatty acids, carotenoids, and proteins, which may help improve animal health by regulating gut microorganisms, supporting intestinal function, and enhancing resistance to environmental stress. This review summarizes current knowledge on how algal biomass and algal-derived preparations influence livestock gut microbiota and discusses their potential roles in improving growth performance, immunity, and sustainable animal production. This review focuses primarily on microalgae and cyanobacteria, while also considering selected macroalgae relevant to livestock feeding. Their effects vary with the source organism, production method, preparation, and animal species, so findings for one product cannot be assumed to apply to another. Overall, algal feed resources represent a promising option for developing healthier and more environmentally friendly livestock systems.

## 1. Introduction

The gastrointestinal tract of livestock harbors a complex and dynamic microbial community that plays a pivotal role in maintaining host health, facilitating nutrient digestion and absorption, and modulating immune responses. The gut microbiota contributes to intestinal barrier homeostasis and influences nutrient utilization, disease susceptibility, and growth performance. Accordingly, disruption of the gut microbial community, commonly referred to as dysbiosis, has been associated with impaired nutrient utilization, increased susceptibility to infection, and dysregulated immune responses across ruminant and monogastric livestock species [1]. These intricate host–microbiota interactions underscore the importance of maintaining a balanced microbial ecosystem for optimal animal productivity and welfare. Historically, antibiotics have been widely used in livestock production for growth promotion, disease prevention, and therapeutic purposes. However, concerns regarding antimicrobial resistance and antibiotic residues in animal-derived products have prompted regulatory and public-health initiatives to reduce the routine use of antibiotics in food-producing animals. Evidence from a systematic review and meta-analysis indicates that interventions restricting antibiotic use in food-producing animals are associated with a reduced prevalence of antibiotic-resistant bacteria in these animals [2]. Consequently, increasing attention has been directed toward nutritional strategies involving probiotics, prebiotics, synbiotics, and other bioactive preparations that may support animal health and production while reducing dependence on antibiotics [3]. This transition is consistent with the broader goal of developing sustainable livestock-production systems that safeguard animal health, public health, and environmental integrity.

In this context, algal biomass has attracted interest as a feed ingredient because of its diverse nutritional and biochemical composition [4,5]. Microalgae and cyanobacteria generally exhibit rapid growth and high biomass productivity, offering considerable potential for scalable biomass production, although their economic competitiveness remains dependent on cultivation or fermentation, harvesting, and downstream processing technologies [5]. Algal feed resources contain a diverse array of bioactive compounds, including polysaccharides, phycobiliproteins, carotenoids, and long-chain polyunsaturated fatty acids such as eicosapentaenoic acid (EPA) and docosahexaenoic acid (DHA) [4,5]. These compounds have been associated with antimicrobial, anti-inflammatory, immunomodulatory, and prebiotic activities and may contribute to the maintenance of gut microbial balance, intestinal function, and host immune responses [5,6]. These properties provide a rationale for evaluating algal biomass and its bioactive components in nutritional strategies that support intestinal health. Growing evidence suggests that dietary inclusion of algal biomass and algal-derived preparations can influence intestinal microbial ecology and host physiology, although the magnitude and direction of these effects depend on the source organism, inclusion level, processing method, and animal species [5]. This review uses “algae” as a practical umbrella term for feed-related applications and focuses primarily on eukaryotic microalgae and cyanobacterial preparations, while also considering selected macroalgae relevant to livestock production. Cyanobacteria are prokaryotes and are distinguished here from eukaryotic algae; preparations described in the cited studies as *Arthrospira* or *Spirulina* are included in this group. This review also includes feed-relevant thraustochytrids, such as *Schizochytrium* and *Aurantiochytrium*, because of their use as DHA sources. These heterotrophic stramenopile protists are distinguished from the microalgae and cyanobacteria discussed here. This review is not restricted to marine organisms. Biomass from microalgae and cyanobacteria, including *Arthrospira*, *Chlorella*, and *Tetraselmis*, has been investigated for its nutritional contribution and potential biological effects in livestock and poultry diets. However, direct evidence for consistent gut microbiota modulation across pigs, poultry, cattle, and sheep remains incomplete. In ovo administration of a *Chlorella polysaccharide* altered cecal microbial composition and predicted microbial metabolic pathways in broiler chickens and increased propionate concentrations [7]. This finding supports an effect of the tested polysaccharide preparation on intestinal fermentation. These studies suggest the potential of algal biomass and its bioactive components to promote intestinal microbial and metabolic homeostasis. Nevertheless, claims regarding improved intestinal barrier integrity, suppression of pro-inflammatory cytokines, and enhancement of systemic immunity should be supported by studies that directly measure these specific outcomes.

Despite these promising outcomes, the practical application of algal biomass and algal-derived preparations in livestock nutrition remains constrained by several challenges. The biochemical composition of algal biomass varies considerably among species and is also influenced by cultivation, harvesting, and processing conditions. Because whole algal biomass contains multiple components, including proteins, polysaccharides, lipids, pigments, minerals, and other bioactive substances, its biological effects cannot always be attributed to a single constituent. Moreover, the digestibility, bioavailability, gastrointestinal transformation, and microbiota-mediated metabolism of many algal compounds remain insufficiently characterized [5]. These uncertainties complicate the formulation of diets containing algal biomass or algal-derived preparations and the selection of appropriate inclusion levels for different livestock species, making it difficult to maximize beneficial effects while minimizing potential adverse outcomes. Interpretation of feeding responses therefore depends on distinguishing the effects of whole biomass from those of individual bioactive components.

Furthermore, the gut microbial communities of livestock are shaped by multiple interacting factors, including diet, host background, environmental conditions, and management practices. For example, seasonal dietary changes exerted a greater influence than host species on the distal gut microbiota of co-grazing yaks and Tibetan sheep, demonstrating the responsiveness of ruminant microbial communities to nutritional and environmental variation [8]. Environmental stressors may also modify the intestinal microbial ecosystem. Heat stress, in particular, has been associated with alterations in gut microbial composition, impaired intestinal barrier integrity, disturbed immune responses, and reduced nutrient transport in livestock and poultry [9]. Consequently, the use of algal biomass and algal-derived preparations in livestock feeding requires careful consideration of animal species, diet, environmental conditions, processing methods, and management practices to optimize gut-health outcomes.

In this review, “algal biomass and algal-derived preparations” is used as a descriptive umbrella term encompassing whole biomass, processed fractions, extracts, and isolated compounds, without implying a regulatory classification. “Feed ingredients” refers to materials incorporated into diets for their nutritional contribution, although they may also exert biological effects. “Dietary supplementation” describes the addition of a preparation to a basal diet in an experimental context and does not imply a distinct regulatory category or marketing authorization. The term “feed additive” is reserved for explicitly regulatory contexts, in which classification and authorization depend on the jurisdiction, product identity, intended function, and conditions of use. Biological activity observed in a feeding trial does not, by itself, establish that a preparation is an authorized feed additive. In summary, the gut microbiota is a major determinant of livestock health and productivity, and its modulation through dietary strategies represents an important avenue for sustainable animal production [1]. The need to reduce routine antibiotic use has accelerated research into nutritional strategies involving algal biomass and algal-derived preparations because of their diverse nutritional and biological properties [5]. Evidence from several livestock and poultry models indicates that dietary microalgae and cyanobacterial preparations may modulate gut microbial composition, support intestinal barrier function, and influence local immune responses; for example, dietary supplementation with *Chlorella vulgaris* altered the cecal microbiome and increased the expression of intestinal barrier- and immune-related markers in broiler chickens [10]. These responses depend on the source organism, composition, inclusion level, processing method, and animal host [5]. This review therefore compares preparation-specific findings, distinguishes observed livestock responses from proposed mechanisms, and evaluates their implications for practical feeding.

Literature-selection approach. Because this article is a narrative rather than a systematic review, the literature was identified through targeted keyword searches and citation chaining rather than a formal PRISMA workflow. Search concepts combined terms for algae, microalgae, cyanobacteria, macroalgae, or individual taxa and preparations with terms for livestock or poultry, gut microbiota, fermentation metabolites, intestinal barrier function, immunity, growth performance, product quality, safety, cultivation, and processing. We prioritized peer-reviewed studies that identified the source organism and preparation and reported outcomes relevant to livestock feeding. Direct in vivo studies in livestock and poultry were given the greatest weight. In vitro fermentation, cell-culture, organoid, rodent, and isolated-compound studies were retained only when they clarified a mechanism or addressed a gap not tested directly in livestock, and their indirectness is identified in the text. Studies were compared according to preparation (whole biomass, extract, fraction, or isolated compound), animal model, dose and delivery route, comparator, and measured endpoint. We did not infer a causal mechanism from taxonomic changes alone, transfer results among algal products or animal species without qualification, or attribute responses to a single constituent when whole biomass was tested. Recent studies were emphasized, while earlier studies were retained when they provided foundational mechanistic evidence or the most direct evidence available. The resulting synthesis is intended to be critical and representative, not exhaustive.

## 2. Major Bioactive Compounds of Algal Feed Resources and Their Physicochemical Properties

### 2.1. Structural Characteristics and Prebiotic Potential of Algal Polysaccharides

Algae synthesize structurally diverse polysaccharides that may occur as intracellular storage compounds, cell-wall components, or extracellular polymers. Depending on the algal species and cultivation conditions, these polymers may include β-glucans and neutral, acidic, or sulfated heteropolysaccharides, with considerable variation in monosaccharide composition, molecular weight, branching patterns, and degree of sulfation [11]. Such structural heterogeneity may affect solubility, resistance to host digestion, and accessibility to microbial enzymes, but the available studies address different preparations and experimental systems. In vitro, purified *Chlorella pyrenoidosa* polysaccharides resisted simulated upper-gastrointestinal digestion and were subsequently fermented by fecal microbiota, with increases in acetate and propionate [12]. A separate in vitro fractionation study showed that neutral and acidic *C. pyrenoidosa* polysaccharide fractions differed in fermentability and in their effects on bacterial taxa and metabolites; enrichment of Bacteroides and Bifidobacterium was specific to one fraction [13]. These two studies therefore link defined polysaccharide properties to distinct fermentation responses, but only under in vitro conditions. By contrast, the piglet study tested 5% whole *Chlorella vulgaris* biomass, not an isolated polysaccharide [14]. It showed changes in community composition and selected taxa, including *Lactobacillus*, without a consistent increase in microbial diversity or short-chain fatty acid concentrations. Because whole biomass also supplies protein, lipids, pigments, minerals, and other components, that response cannot be assigned specifically to polysaccharides. The current evidence thus supports structure-dependent fermentation of selected isolated *Chlorella polysaccharides*, but it does not establish that whole *Chlorella* biomass acts through its polysaccharide fraction or that algal preparations generally increase overall microbial diversity or SCFA production in vivo. Taxon abundance, community diversity, and fermentation output should therefore be treated as separate endpoints.

### 2.2. Antimicrobial and Immunomodulatory Effects of Polyunsaturated Fatty Acids

Microalgae and feed-relevant thraustochytrids are important sources of long-chain omega-3 polyunsaturated fatty acids, particularly eicosapentaenoic acid (EPA, C20:5 n-3) and docosahexaenoic acid (DHA, C22:6 n-3), and have attracted increasing interest as sustainable sources of these compounds for food and feed applications [15]. Free EPA and DHA have demonstrated antibacterial activity against several foodborne bacterial species under in vitro conditions, including *Salmonella enteritidis* and *Salmonella typhimurium* [16]. Their antibacterial effects may involve perturbation of bacterial membrane structure and function; however, direct evidence that dietary algae-derived EPA and DHA reduce pathogen colonization in the livestock gastrointestinal tract remains limited. Beyond their potential direct antimicrobial effects, EPA and DHA modulate host inflammatory responses through several mechanisms, including alterations in cell-membrane phospholipid composition, activation of peroxisome proliferator-activated receptor gamma (PPARγ), inhibition of nuclear factor kappa B (NF-κB) activation, and generation of inflammation-resolving lipid mediators [17]. Evidence from porcine intestinal epithelial cells further indicates that EPA and DHA can protect tight-junction integrity through both PPARγ-dependent and PPARγ-independent mechanisms, including preservation of claudin-4 and zonula occludens-1 under mycotoxin-induced stress [18]. The evidence distinguishes two activities of EPA and DHA: direct antibacterial effects in vitro and modulation of inflammatory and epithelial responses in mechanistic models. Protection of tight-junction integrity in porcine epithelial cells provides support for a barrier-related action under mycotoxin exposure, but it does not establish reduced intestinal pathogen colonization in livestock. Together, these findings support the immunomodulatory potential of EPA and DHA but do not establish their effectiveness as antibiotic substitutes in livestock.

### 2.3. Antioxidant Activities of Carotenoids and Phycobiliproteins

Certain microalgae and cyanobacteria are important sources of antioxidant pigments, including carotenoids such as astaxanthin and β-carotene and phycobiliproteins such as phycocyanin. These compounds can exert antioxidant effects through direct scavenging of reactive oxygen species, inhibition of lipid peroxidation, and enhancement of endogenous antioxidant defenses, although their activities vary according to compound structure, concentration, source organism, and experimental conditions [19]. In heat-stressed broiler chickens, dietary supplementation with astaxanthin derived from *Haematococcus pluvialis* increased the expression of cytoprotective genes, including *SOD2*, *GPX3*, and *TXN*, as well as epithelial integrity-related genes such as *CLDN1* and *MUC2*, suggesting that astaxanthin may help protect the intestinal epithelium against oxidative injury [20]. In broiler chickens, dietary phycocyanin extracted from *Arthrospira platensis* improved antioxidant indices, including those related to superoxide dismutase, catalase, glutathione, and total antioxidant capacity, and reduced malondialdehyde concentrations [21]. The broiler studies provide direct evidence of antioxidant-related responses to the tested astaxanthin and phycocyanin preparations. Astaxanthin altered cytoprotective and epithelial integrity-related gene expression under heat stress, whereas phycocyanin improved measured antioxidant indices and reduced lipid peroxidation. Thus, the clearest conclusion concerns antioxidant responses in poultry; these findings do not by themselves establish Nrf2-dependent signaling or microbiota-mediated improvements in growth and welfare.

## 3. Effects of Algal Biomass and Algal-Derived Preparations on Livestock Gut Microbial Communities

### 3.1. Changes in Potentially Beneficial Bacterial Populations

Algal biomass and isolated polysaccharide fractions may influence gut microbial composition and fermentation by providing microbiota-accessible carbohydrate substrates. However, these effects depend on the structural characteristics of the polysaccharides, the source organism, the inclusion level, processing conditions, and the animal host. Microbial utilization of these carbohydrates may alter the production of organic acids and other fermentation metabolites, thereby modifying the intestinal environment, but selective enrichment of specific beneficial bacteria should not be assumed for all algal polysaccharides. In broiler chickens, dietary supplementation with 0.25–1.0% *Spirulina* (*Arthrospira platensis*) produced a dose-dependent increase in the cecal *Lactobacillus* population within the tested inclusion range [22]. In weaned piglets, dietary inclusion of 5% *Chlorella vulgaris* altered the fecal bacterial community and increased the abundance of selected taxa, including *Lactobacillus*, although volatile fatty acid concentrations did not increase consistently [14]. In broiler chickens, in ovo administration of a *Chlorella polysaccharide* also altered the cecal microbial community, predicted microbial metabolic pathways and fermentation metabolites, including propionic acid [7]. An increase in *Lactobacillus* abundance is a shared observation in the cited dietary Spirulina and *Chlorella* studies, despite differences in the preparation, inclusion level, and animal model. Fermentation responses were less uniform, indicating that enrichment of a selected bacterial group does not necessarily translate into increased SCFA production. The in ovo polysaccharide study additionally demonstrates a response to a defined fraction, but its administration route makes it distinct from dietary biomass supplementation.

### 3.2. Competitive Exclusion and Direct Inhibition of Pathogenic Bacteria

Algal biomass and extracts may limit intestinal pathogens through both direct antimicrobial activity and indirect modulation of beneficial microbial populations. In vitro screening showed that extracts from *Isochrysis galbana*, *Scenedesmus* sp. NT8c, and *Chlorella* sp. FN1 preferentially inhibited selected Gram-positive bacteria. Fractionation and chemical characterization identified linoleic acid, oleic acid, eicosapentaenoic acid, and docosahexaenoic acid as candidate antimicrobial constituents, indicating that the antibacterial activity of microalgae is compound- and bacterial-strain-dependent [23]. Accordingly, these findings should not be generalized as evidence that all algal extracts consistently inhibit major livestock pathogens such as *Salmonella* spp. and *Clostridium perfringens*.

Algae may also indirectly restrict pathogen establishment by supporting potentially beneficial intestinal bacteria. In broiler chickens, dietary supplementation with 0.25–1.0% Spirulina (*A. platensis*) produced a dose-dependent increase in the cecal *Lactobacillus* population [22]. However, this observation alone does not demonstrate direct competitive exclusion or reduced pathogen colonization. More direct evidence of a cooperative microalga–probiotic interaction has been obtained in vitro: the marine microalga *Isochrysis galbana* supported the growth of *Lactobacillus reuteri*, and the fermented combination reduced the adhesion of adherent-invasive *E. coli* to intestinal epithelial cells and its invasion of these cells more effectively than either component alone [24]. These studies support different levels of inference: extract screening demonstrates strain-selective antibacterial activity, the microalga–probiotic experiment demonstrates reduced epithelial adhesion and invasion in vitro, and the broiler feeding study demonstrates enrichment of *Lactobacillus*. Only the first two directly assess pathogen-related endpoints. Consequently, the current evidence supports selected antimicrobial and anti-adhesion activities, but increased abundance of beneficial bacteria alone is not evidence of competitive exclusion or antibiotic-equivalent protection.

### 3.3. Remodeling of Gut Microbial Metabolites

Dietary algae and their isolated polysaccharide fractions may alter the metabolic activity of the gut microbiota, including the production of short-chain fatty acids (SCFAs). However, the direction and magnitude of these changes depend on the algal species, bioactive fraction, dose, delivery method, and animal host. In broiler chickens, in ovo administration of a *Chlorella* polysaccharide altered the cecal microbial community and predicted microbial metabolic pathways and increased the concentration of propionic acid, providing direct evidence that a defined algae polysaccharide can modulate intestinal microbial fermentation [7]. Nevertheless, current livestock studies do not demonstrate a consistent increase in butyrate following algal supplementation. Butyrate is an important microbial metabolite that contributes to colonocyte energy metabolism, intestinal barrier maintenance, and mucosal immune regulation [25]. Therefore, increases in butyrate, when directly demonstrated, may provide a plausible link between algae-induced microbial fermentation and improved intestinal homeostasis.

Gut microorganisms also participate in the deconjugation and transformation of primary bile acids into a diverse range of secondary bile acids, some of which signal through receptors such as the farnesoid X receptor (FXR) and thereby influence host lipid, glucose, and bile acid metabolism [26]. However, direct evidence that algal sulfated polysaccharides specifically promote deoxycholic acid production or reduce fatty liver risk through FXR signaling in livestock remains insufficient. Among the examples reviewed, altered fermentation is supported by a measured increase in propionate after in ovo administration of a *Chlorella* polysaccharide. By comparison, the proposed bile acid–FXR mechanism lacks direct evidence from livestock receiving algal preparations. The evidence therefore supports preparation- and metabolite-specific changes, rather than a general increase in beneficial microbial metabolites. SCFA production and bile acid signaling should consequently be treated as separate evidence streams when interpreting downstream host responses (Figure 1).

## 4. Potential Host Pathways Associated with the Effects of Algal Bioactive Compounds on Livestock Gut Health

The mechanistic evidence discussed in this section varies substantially in its directness and biological context. For clarity, evidence obtained from livestock or poultry receiving algal preparations and involving direct measurement of the proposed pathway is described as direct livestock evidence. Findings from rodent, cell-culture, organoid, or non-algal intervention studies are treated as indirect or supportive evidence for mechanisms in livestock receiving algal preparations. Studies of isolated compounds are interpreted according to the animal model and measured endpoints and are not assumed to explain responses to whole biomass. Mechanistic links that have not been directly tested in algae-fed livestock are presented as proposed or hypothetical pathways and should not be interpreted as established causal mechanisms.

### 4.1. Potential Involvement of TLR- and NLR-Related Signaling

Algal bioactive compounds may modulate innate immune signaling, although the direction and magnitude of their effects depend on the bioactive compound, dose, inflammatory context, and experimental model. Current evidence does not support a universal mechanism whereby algal β-glucans and phycocyanin are directly recognized by TLR2 or TLR4 on intestinal macrophages and dendritic cells in livestock. In a mouse model of radiation-induced acute intestinal injury, phycocyanin alleviated intestinal damage in association with modulation of the gut microbiota and attenuation of the TLR4/MyD88/NF-κB signaling pathway [27]. However, this finding indicates pathway modulation rather than direct binding of phycocyanin to TLR4. Separately, an extract of Spirulina maxima, rather than purified phycocyanin, suppressed LPS-induced activation of the NLRP3 inflammasome in murine and human macrophages. The extract reduced ERK phosphorylation and ROS generation, attenuated NF-κB signaling, and decreased NLRP3 expression, caspase-1 activation, and the maturation of IL-1β and IL-18 [28]. These findings provide mechanistic evidence for the anti-inflammatory activity of a Spirulina preparation but do not demonstrate protection against *Salmonella enterica* infection or confirm the same mechanism in the livestock intestine.

Livestock-level evidence remains comparatively limited. In broiler chickens fed a low-protein diet, supplementation with 100 g/kg *Spirulina platensis* reversed increases in circulating pro-inflammatory cytokines, NLRP3 expression, and bacterial translocation to the liver. Nevertheless, the study did not demonstrate direct intestinal binding to TLRs or direct inhibition of NLRP3 by an isolated algal compound [29]. Across these models, changes in inflammatory signaling and cytokine-related endpoints support an anti-inflammatory response to the tested preparations. However, the preparations and experimental contexts differ: purified phycocyanin, a Spirulina extract, and whole Spirulina biomass cannot be treated as mechanistically interchangeable. These interventions modified inflammatory responses under the tested conditions, but the findings do not identify direct receptor binding as a shared mechanism.

### 4.2. Evidence for Effects on Intestinal Barrier Function

The integrity of the intestinal barrier is essential for maintaining gut homeostasis by restricting the translocation of luminal microorganisms and microbial products while supporting nutrient absorption and mucosal immune function. Livestock studies report changes in intestinal barrier markers following supplementation with whole algal biomass or algal-derived carotenoids. Evidence linking these responses to RhoA/ROCK or AMPK-mTOR signaling is limited. In broiler chickens, dietary supplementation with *Chlorella vulgaris* increased the expression of the tight-junction protein occludin and the antimicrobial peptide avian β-defensin 5, together with an increase in cecal microbial richness, indicating an association between algal supplementation and changes in markers of epithelial defense [10]. However, this study did not establish ROCK- or AMPK-dependent signaling as the underlying mechanism.

Algae-derived astaxanthin may reinforce both tight-junction- and mucus-associated components of the intestinal barrier. In heat-stressed broilers, astaxanthin supplementation upregulated epithelial integrity-related genes, including *CLDN1*, *LOX*, and *MUC2*, as well as cytoprotective genes associated with antioxidant defense [20]. Consistently, in broilers challenged with *Salmonella enteritidis*, astaxanthin improved ileal morphology and antioxidant capacity and increased the expression of ZO-1 and MUC2, accompanied by changes in the composition of the cecal microbiota [30]. These findings suggest that astaxanthin can protect intestinal epithelial integrity under oxidative and enteric stress. Nevertheless, direct stimulation of goblet-cell secretory activity and an increase in mucus-layer thickness have not yet been conclusively demonstrated in livestock.

The broiler studies converge on changes in epithelial integrity-related markers: *Chlorella* supplementation increased occludin expression, while astaxanthin affected tight-junction- and mucus-associated genes and improved intestinal morphology under challenging conditions. This convergence supports an effect on epithelial maintenance in the tested poultry models. However, marker expression and morphology are not interchangeable with direct measurements of permeability or mucus secretion, and the observed responses do not identify a common receptor or kinase pathway (Figure 2).

### 4.3. Potential Involvement of IGF-1 and Microbiota–Gut–Brain Signaling in Growth Regulation

Algal supplementation can influence nutrient utilization, intestinal physiology, and, under some dietary conditions, growth performance in livestock. However, direct evidence that these responses are mediated by insulin-like growth factor-1 (IGF-1) or microbiota–gut–brain signaling remains limited. In weaned piglets, dietary inclusion of 5% *Chlorella vulgaris* increased duodenal villus height and altered the abundance of several bacterial taxa, including *Lactobacillus*, but did not significantly affect the average daily gain or feed conversion ratio. Moreover, the effects on cecal volatile fatty acid concentrations were not uniform across treatments [14]. These findings support microbiota and intestinal modulation by microalgae but do not establish a downstream endocrine or neuroendocrine mechanism.

Evidence directly linking algal supplementation to circulating IGF-1 is also inconclusive. In broiler chickens, dietary supplementation with *Spirulina platensis* extract improved final body weight, body-weight gain, and feed conversion ratio, whereas serum IGF-1 concentrations did not differ significantly among treatments [31]. Therefore, the observed improvement in growth performance cannot be specifically attributed to activation of the IGF-1 pathway. Other mechanisms, including changes in nutrient availability, antioxidant status, intestinal development, and immune function, may also contribute to the productive responses to algal supplementation.

Mechanistic studies in other experimental models suggest possible explanations for these responses. Short-chain fatty acids can stimulate glucagon-like peptide-1 secretion from intestinal L-cells through free fatty acid receptors, particularly FFAR2, although this evidence does not directly demonstrate enhanced GLP-2 secretion following algal supplementation [32]. In mice, GLP-2 increased intestinal IGF-1 expression and secretion and required IGF-1 signaling for part of its intestinal trophic activity [33]. This represents a local intestinal GLP-2-IGF-1 mechanism rather than a demonstrated pathway in which GLP-2 directly stimulates hepatocytes to increase systemic IGF-1 production.

Microbiota-dependent neural signaling is likewise biologically plausible but has not been directly demonstrated in livestock receiving algal biomass or algal-derived preparations. For example, administration of *Lactobacillus rhamnosus* altered central GABA-receptor expression and reduced stress-related responses in mice through a vagus-dependent mechanism [34]. Nevertheless, this evidence cannot be directly extrapolated to microalgae-induced microbiota changes in farm animals. Consequently, effects of microalgae on vagal signaling, serotonin or GABA metabolism, hypothalamic–pituitary–adrenal axis activity, appetite, and stress-induced anorexia should currently be presented as proposed mechanisms requiring experimental validation.

Intestinal, growth, and endocrine responses did not consistently occur together in the cited studies. Chlorella altered intestinal morphology and microbial composition without improving growth, whereas Spirulina extract improved broiler growth without significantly changing circulating IGF-1. These findings show that intestinal responses do not necessarily predict productive responses and that improved growth cannot be attributed to increased circulating IGF-1 on the basis of the available measurements. The SCFA–GLP-2–IGF-1 and microbiota–vagus–brain pathways therefore provide explanatory hypotheses rather than demonstrated mechanisms of the reported livestock responses (Figure 2). The major algal bioactive components, their reported effects on gut microbiota, and associated host responses are summarized in Table 1.

## 5. Application Effects and Challenges of Algal Biomass and Algal-Derived Preparations in Livestock and Poultry Production

### 5.1. Evidence from Pig and Poultry Studies: Dose- and Product-Dependent Responses

The studies differ in algal source, product composition, inclusion level, administration route, treatment duration, diet formulation, animal age, and measured outcomes. Given this heterogeneity, we compare reported doses and responses qualitatively rather than estimating a pooled dose–response relationship. The summarized results should be interpreted as product- and context-specific observations rather than as evidence of a universally applicable dose–response relationship.

The effects of dietary Spirulina on pig and poultry production are strongly influenced by the inclusion level, animal age, diet formulation, amino acid balance, and processing or enzyme strategy, and therefore do not follow a universal linear dose–response relationship. In weaned piglets, dietary inclusion of 1% Spirulina increased gross-energy digestibility and tended to improve the digestibility of dry matter, organic matter, and neutral detergent fiber, but did not significantly improve growth performance or reduce diarrhea incidence [35]. In a separate study, oral administration of Spirulina around weaning reduced the occurrence of post-weaning diarrhea and increased body-weight gain during the suckling period [36]. This study used oral administration at approximately 385 mg/kg body weight per day; its findings therefore apply to that regimen and cannot be directly translated into a dietary inclusion percentage. At a higher dietary inclusion level, the responses were less favorable. Incorporation of 10% Spirulina into post-weaning piglet diets reduced final body weight and average daily gain and impaired feed conversion, accompanied by increased digesta viscosity and reduced crude-protein digestibility [37]. Neither a commercial multi-carbohydrase preparation nor lysozyme fully restored growth performance. These findings suggest that the adverse effects of high Spirulina inclusion are principally associated with digestive constraints, including the resistance of algal proteins to endogenous peptidases, rather than a demonstrated reduction in palatability caused by algal odor or taste. The adverse response at 10% inclusion is specific to the tested preparation and diet and does not define a general upper inclusion limit for Spirulina. Proteomic findings also require cautious interpretation. Dietary inclusion of 10% Spirulina was associated with changes in muscle glycogen metabolism and the utilization of endogenous nutrient reserves, whereas lysozyme supplementation altered the abundance of proteins related to the contractile apparatus and protein synthesis [38]. These molecular changes indicate metabolic adaptation but do not directly demonstrate enhanced nutrient assimilation, muscle hypertrophy, or increased lean-tissue deposition. Integrated intestinal proteomic, metabolomic, and histological analyses identified changes in pathways related to glucose uptake and protein synthesis. These changes occurred alongside increased digesta viscosity and no significant improvement in intestinal morphology [39]. Comparable digestive constraints have been observed in poultry. In broiler chickens, 15% dietary Spirulina reduced body-weight gain and worsened feed conversion through increased digesta viscosity, probably associated with the gelation of poorly digested algal proteins [40]. Lysozyme disrupted the Spirulina cell wall, whereas the commercial multienzyme preparation did not, indicating that enzyme efficacy is highly substrate-specific. Overall, the peptidoglycan-rich cell wall and resistant protein matrix of Spirulina can limit nutrient accessibility at high inclusion levels. Enzyme-assisted processing remains a promising strategy, but its effectiveness depends on enzyme specificity, algal inclusion level, diet composition, and the simultaneous degradation of both cell-wall polymers and resistant proteins [41].

In broiler chickens, dietary supplementation with 2% DHA-rich microalgae increased breast-muscle n-3 polyunsaturated fatty acids, while additional methionine reduced white striping and myopathic lesions without impairing growth performance [42]. However, high dietary inclusion of DHA-rich algal preparations may impair growth performance. Supplementation with 4% *Aurantiochytrium acetophilum*, providing 6.8 g DHA/kg diet, reduced body-weight gain, feed efficiency, and breast-muscle weight and increased lipid oxidation [43]. Dietary inclusion of 10% *Chlorella vulgaris* did not compromise broiler growth and improved meat yellowness, carotenoid content, and tenderness, although CAZyme supplementation provided no consistent additional benefit [44]. A meta-analysis also showed that seaweed supplementation improved broiler weight gain and feed conversion, although these macroalgal findings should not be directly generalized to microalgae [45]. In laying hens, 1–3% *Nannochloropsis limnetica* dose-dependently increased egg-yolk EPA and DHA, while 2–3% inclusion improved the laying rate without adversely affecting egg quality or nutrient retention [46]. Across these studies, changes in product composition, particularly n-3 fatty acid enrichment, are more directly supported than a general growth-promoting effect. The adverse response to 4% *Aurantiochytrium* and the absence of growth impairment with 10% *Chlorella* also show why inclusion percentages cannot be compared independently of product composition and diet formulation. Algal preparations should therefore be selected for a defined nutritional or product-quality objective, with growth performance and oxidative stability evaluated as separate outcomes.

### 5.2. Special Considerations in Ruminants

The use of algae in ruminant diets is constrained by ruminal biohydrogenation, which converts part of the EPA and DHA into more saturated fatty acids and reduces their transfer to meat and milk. Rumen-protected or encapsulated formulations may improve the post-ruminal delivery of these fatty acids and enhance their deposition in animal products [47]. Inclusion level also influences the response of the rumen microbial community. High inclusion levels of DHA-rich *Schizochytrium* spp. can alter the rumen microbial community, including cellulolytic bacteria such as *Fibrobacter succinogenes*, and may consequently disturb fiber fermentation [48]. Therefore, both inclusion level and delivery strategy should be optimized to improve PUFA utilization without disrupting rumen function.

A particularly promising approach involves the use of the red macroalga *Asparagopsis taxiformis*, which accumulates bromoform, a halogenated methane analog that suppresses the terminal step of archaeal methanogenesis by interfering with methyl-coenzyme M reductase-associated pathways. In beef steers, dietary supplementation with *A. taxiformis* reduced methane yield by 45–68% across inclusion levels and by up to 80% under low-forage dietary conditions, although the magnitude of the response varies considerably with diet composition, inclusion rate, and bromoform concentration [49,50]. These trials establish a substantial methane-reducing effect of the tested *A. taxiformis* preparations in beef cattle. Variation with forage level, inclusion rate, and bromoform concentration identifies the preparation-diet combination as a determinant of efficacy. The trials therefore support the methane-reducing efficacy of the tested preparations. Suitability for routine feeding also depends on their effects on feed intake, iodine and bromide exposure, and long-term animal health.

### 5.3. Commercial Feasibility, Product Stability, and Cost–Benefit Considerations

The commercial viability of algal biomass and algal-derived preparations for livestock feeding depends on production cost, product stability, and consistency among production batches. Biomass harvesting, dewatering, and drying remain major cost drivers, with biomass recovery accounting for approximately 20–30% of total production costs in some production systems [51]. Processing conditions may also affect product quality. For example, thermal exposure during spray drying may reduce the stability of thermolabile pigments such as phycocyanin [52]. Enzymatic pretreatment can improve the recovery of proteins and oils from algal biomass [53,54], but its commercial value depends on enzyme cost, process scalability, recovery efficiency, and retention of sensitive bioactive compounds. In this section, these processes are considered primarily in relation to commercial feasibility and their influence on the quality of livestock-feed products; their technical principles and optimization are discussed separately in Section 8.

The storage stability of sensitive bioactive compounds is another important practical consideration. Astaxanthin is susceptible to degradation under elevated temperature, oxygen exposure, and illumination, whereas microencapsulation can improve its retention during storage [55]. Integration of microalgal cultivation with appropriately treated nutrient-rich waste streams may reduce external nutrient inputs and generate protein-rich biomass for animal feed [56]. Commercial production requires contaminant control, stable cultivation conditions, and consistent product composition across batches. Cost–benefit assessments should therefore balance potential animal responses against the additional costs of cultivation, processing, stabilization, formulation, and quality control. These considerations determine whether the bioprocess technologies described in Section 8 can be translated into safe, standardized, and economically viable feed products. An overview of the application effects, molecular mechanisms, and challenges of algal biomass and algal-derived preparations in livestock production is provided in Table 2, while dose- and product-dependent responses in livestock and poultry are summarized in Table 3.

## 6. Current Research Limitations and Future Directions

The evidence reviewed above identifies three priorities that directly affect interpretation and application: linking product composition and processing to the release and bioavailability of active components, determining whether microbial and molecular changes translate into functional animal outcomes, and defining product-specific efficacy–safety trade-offs. These priorities address the main sources of disagreement among studies and provide a framework for the research directions discussed below.

### 6.1. Unclear In Vivo Metabolism and Delivery Mechanisms of Active Components

Despite the potential of algae to modulate livestock gut microbiota, current evidence on the gastrointestinal fate and bioavailability of their active components remains limited, with many studies relying on in vitro digestion models or isolated compounds [57]. The complex cell-wall structure of microalgae can restrict the release of intracellular proteins, lipids, pigments, and other bioactive substances, while the digestion and microbial fermentation of marine algal polysaccharides remain insufficiently characterized in vivo [58]. Moreover, differences in digestive physiology and gut microbial composition among livestock species may affect compound release, fermentation, and biological responses, but systematic species-specific comparisons remain scarce. Future studies could combine tracing of labeled algal components with metabolomics, microbial community sequencing, and single-cell imaging. Fluorescent glycan probes have been used to directly visualize polysaccharide uptake by intestinal bacteria, while metabolic labeling coupled with fluorescence-activated cell sorting can identify specific glycan-utilizing bacterial populations [59,60]. These approaches could reveal where and when algal components are degraded, which microorganisms utilize them, and how these processes affect their availability to the host.

### 6.2. Heavy Metal Accumulation and Metabolic Interference

The safety of long-term algal supplementation requires careful evaluation, particularly with respect to metal contamination and excessive lipid intake. Microalgae can adsorb toxic elements onto their surfaces and accumulate them within cells. The accumulation of elements such as arsenic, cadmium, and lead varies with species, environmental concentrations, pH, and cultivation conditions [61]. The detection of toxic metals in commercial algae products further emphasizes the need for strict control of cultivation water, nutrient sources, and processing conditions [62]. However, direct evidence of metal transfer from algae feed to edible livestock tissues remains limited. Risk management should therefore prioritize strain selection, contaminant-free cultivation systems, and routine batch testing rather than costly chromatographic detoxification. Algal n-3 PUFAs can modify tissue fatty acid profiles and prostaglandin metabolism, which may influence reproductive physiology. Nevertheless, current evidence does not demonstrate that a reduced n-6/n-3 ratio consistently impairs fertility in livestock, and several reproductive outcomes remain insufficiently studied [63]. Long-term dose–response and multigenerational feeding trials are therefore needed to establish safe inclusion levels and assess contaminant transfer, metabolic responses, and reproductive performance. Such evaluations are essential for the safe and consistent application of microalgae in animal feed [64].

## 7. Potential Host-Intrinsic Molecular Responses to Algal Bioactives

### 7.1. Potential Epigenetic Regulation Associated with Algal Bioactives and Microbial Metabolites

Algal supplementation may influence gene regulation in the intestinal epithelium through microbial metabolites, although direct evidence of epigenomic changes in livestock is limited. Dietary supplementation with *Phaeodactylum tricornutum* increased intestinal acetate, propionate, and butyrate production in mice, supporting a link between algae intake and microbial SCFA generation [65]. Among these metabolites, butyrate functions as an HDAC inhibitor and enhances histone H3 acetylation at regulatory regions of the *Foxp3* locus, thereby promoting colonic regulatory T-cell differentiation and intestinal immune homeostasis [66]. Butyrate may also strengthen epithelial barrier function through HDAC-dependent induction of IL-10 receptor expression and repression of the permeability-promoting tight-junction protein claudin-2 [67]. Astaxanthin has demonstrated epigenetic activity in cultured human prostate cells by reducing *GSTP1* promoter methylation and inhibiting DNMT and HDAC activities. However, it did not significantly alter methylation of the *Nrf2* promoter, and comparable effects have not been confirmed in intestinal epithelial cells or livestock models [68]. Similarly, miR-146a can attenuate experimental intestinal inflammation by targeting TRAF6 and IRAK1 and suppressing NF-κB signaling [69], but there is currently insufficient evidence that algal polysaccharides or β-glucans directly regulate miR-146a or miR-155 in the livestock intestine. The strongest mechanistic evidence discussed here concerns butyrate-dependent histone regulation in non-livestock models, whereas the astaxanthin findings demonstrate tissue- and gene-specific effects in cultured cells. These results establish the plausibility of epigenetic regulation but do not connect algal feeding to epigenetic changes in the livestock intestine. Epigenetic mechanisms therefore serve as a framework for interpreting candidate pathways, rather than as an established explanation for the feeding responses reviewed here (Figure 3).

### 7.2. Potential Modulation of Intestinal Stem-Cell Activity and Epithelial Renewal

The intestinal epithelium undergoes continuous renewal driven by Lgr5-positive intestinal stem cells located at the crypt base [70]. Although algal supplementation may influence epithelial renewal through microbial metabolites and lipid-derived bioactives, direct evidence linking whole algal biomass to intestinal stem-cell regulation remains limited. Butyrate should not be regarded as a universal promoter of ISC proliferation. Differentiated colonocytes normally metabolize luminal butyrate and protect crypt-base stem cells from direct exposure. When epithelial injury disrupts this spatial barrier, butyrate can suppress stem and progenitor cell proliferation through HDAC inhibition and FOXO3-dependent transcriptional regulation [71]. Butyrate may nevertheless support intestinal homeostasis indirectly through GPR109A signaling, which induces anti-inflammatory properties in macrophages and dendritic cells and promotes the differentiation of regulatory and IL-10-producing T cells [72]. Experiments with purified EPA provide mechanistic evidence linking this fatty acid to ISC regulation. Algal lipid EPA has been shown to promote ISC proliferation and differentiation and enhance colonic epithelial regeneration through the GPR120-LSD1-WNT signaling pathway in intestinal organoids and mouse colitis models [73]. However, comparable ISC-specific effects of algal DHA, resolvins, or whole algal preparations remain to be demonstrated. Dietary microalgae improved intestinal morphometry in broiler chickens and increased the villus height-to-crypt depth ratio in weaned piglets [74,75]. These tissue-level responses were observed directly in livestock, whereas the evidence for ISC regulation comes from separate mechanistic models. The suppression of stem-cell proliferation by butyrate under epithelial injury and the promotion of regeneration by EPA in organoid and mouse models demonstrate that these compounds do not share a uniformly stimulatory effect on ISCs. Accordingly, improved villus morphology should be interpreted as a tissue-level response, rather than as evidence of a specific stem-cell mechanism (Figure 3).

### 7.3. Potential Systemic Metabolic Regulation via the Gut–Liver and Gut–Adipose Axes

Algal supplementation may influence systemic metabolism through interactions among the gut, liver, and adipose tissue, although direct mechanistic evidence in livestock remains limited. Propionate produced by microbial fermentation is an important substrate for hepatic gluconeogenesis, particularly in ruminants, and therefore contributes to glucose homeostasis [76]. Butyrate has also been shown to suppress hepatic lipogenesis and alleviate steatosis through the LKB1-AMPK-Insig pathway in mouse and hepatocyte models; however, this effect has not been directly demonstrated for microalgae-derived butyrate in livestock [77]. Algal EPA and DHA may additionally affect adipose tissue metabolism, but their actions are context-dependent. In cultured adipocytes, DHA increased PPARγ expression and adiponectin secretion, suggesting a potential role in improving insulin sensitivity [78]. Nevertheless, evidence that algae PUFAs consistently reduce adipocyte hypertrophy or suppress Wnt/β-catenin signaling in livestock is insufficient. SCFAs can also stimulate GLP-1 secretion from intestinal L cells through FFAR2 signaling, providing a potential connection between microbial fermentation and host glucose regulation [32]. Whether algal supplementation induces comparable changes in GLP-1 or PYY secretion in livestock remains unclear. In sheep fed high-energy diets, dietary supplementation with *Schizochytrium* sp. altered liver and muscle fatty acid profiles and the expression of genes involved in lipid metabolism [79]. These outcomes establish a metabolic response to the tested preparation but do not distinguish direct effects of its nutrient composition from microbiota-mediated signaling. These findings support an effect on lipid metabolism, but the proposed gut–liver and gut–adipose mechanisms draw on evidence from different experimental models and have not been demonstrated as integrated pathways in livestock (Figure 3).

## 8. Technical Advances in the Production and Processing of Algal Biomass and Algal-Derived Preparations for Livestock Feeding

### 8.1. Technical Advances in Cultivation and Harvesting

Scaling up the production of algal biomass and algal-derived preparations for livestock feeding requires increased biomass and target-compound productivity while controlling energy and downstream processing costs. Advanced photobioreactors, particularly flat-panel and airlift configurations, can improve light distribution, gas transfer, and culture mixing. However, their effects on astaxanthin or PUFA accumulation remain species- and condition-dependent rather than universally beneficial [80]. Two-stage cultivation is particularly suitable for *Haematococcus pluvialis*, in which biomass is first accumulated under growth-favoring conditions, and astaxanthin synthesis is subsequently induced by controlled nutrient, light, or salinity stress [81]. Heterotrophic and mixotrophic cultivation can reduce dependence on illumination and enable relatively high biomass densities. Optimized glucose-based fermentation, for example, increased biomass and DHA production by *Schizochytrium* sp. [82]. Nevertheless, its economic advantage depends on carbon-source price, oxygen demand, contamination control, and reactor operation. Harvesting remains a major production bottleneck. Electrocoagulation–flotation can provide rapid biomass concentration, but electrode consumption, metal residues, and operating conditions must be considered when the recovered biomass is intended for feed use [83]. Magnetic separation is another promising approach, although its scalability depends on the cost, biocompatibility, recovery, and reuse of magnetic particles [84]. Integration with pretreated livestock-waste digestate may reduce the demand for synthetic nitrogen and phosphorus while generating protein-rich biomass. However, current life-cycle assessments do not consistently demonstrate an economic or environmental advantage over conventional feed ingredients [85]. Wastewater-derived biomass must therefore be monitored for pathogens, toxic metals, veterinary drug residues, and batch-to-batch compositional variability before feed application. These technologies should be evaluated jointly for productivity, safety, energy use, and economic feasibility, rather than for biomass yield alone.

### 8.2. Technical Advances in Cell-Wall Disruption and Bioactive Compound Extraction

The rigid and compositionally diverse cell walls of many microalgae restrict the release and bioavailability of intracellular proteins, lipids, pigments, and other bioactive compounds. Effective disruption methods must therefore balance extraction efficiency, energy consumption, and the preservation of oxidation- and heat-sensitive components. Mechanical methods such as high-pressure homogenization, bead milling, and ultrasonication are widely used for algal cell disruption. High-pressure homogenization and bead milling achieved more than 95% cell disintegration in *Nannochloropsis gaditana*, although soluble-protein recovery remained substantially lower than the degree of cell disruption [86]. These treatments may also generate heat and expose PUFAs and pigments to oxidation, requiring temperature control and short processing times. Pulsed electric field (PEF) treatment uses membrane electroporation to facilitate the release of cellular components while limiting thermal exposure. In *Chlorella vulgaris*, PEF enhanced selective protein release, but high treatment intensities adversely affected subsequent cell growth, indicating that process conditions must be optimized for each strain and target compound [87]. Enzyme-assisted treatment can degrade susceptible cell-wall components under relatively mild conditions. Combined sonication and enzymatic hydrolysis increased carbohydrate and soluble-protein recovery from algal biomass, with recovery depending on enzyme dosage and treatment conditions [88]. Following cell disruption, extraction conditions should be selected according to compound polarity and stability. Supercritical CO_2_, with ethanol as a co-solvent, achieved high astaxanthin recovery from disrupted *Haematococcus pluvialis* cells under laboratory-scale conditions [89]. However, pretreatment requirements, pressure-related equipment costs, and scale-up performance must be considered before feed-grade application. For water-soluble phycobiliproteins, aqueous two-phase extraction increased C-phycocyanin purity while maintaining a relatively high recovery yield [90]. Overall, downstream technologies should be evaluated according to recovery yield, residual processing agents, oxidative stability, energy demand, scalability, and feed-safety requirements rather than extraction efficiency alone.

## 9. Conclusions

The studies reviewed support several distinct applications of algal biomass and algal-derived preparations in livestock nutrition. Selected lipid-rich preparations enrich animal products with n-3 fatty acids, while *Asparagopsis taxiformis* reduces enteric methane in beef cattle under the tested conditions. Selected poultry studies also report improved antioxidant indices and changes in markers of epithelial integrity. These findings support specific uses of characterized preparations rather than a general growth-promoting effect of algae.

Product composition, inclusion level, processing, and diet formulation influence the balance between benefits and adverse effects. High Spirulina inclusion impaired performance in the cited piglet and broiler studies, whereas selected lower-dose interventions affected digestibility or diarrhea without consistently improving growth. Lipid-rich preparations likewise altered tissue fatty acid composition, but higher inclusion could impair performance or oxidative stability. Product quality, growth, and tolerability should therefore be assessed as separate outcomes.

Mechanistic interpretation should distinguish directly measured animal responses from proposed causal pathways. Changes in microbial composition, fermentation metabolites, antioxidant indices, and epithelial markers do not by themselves establish a causal sequence leading to improved performance. Integrated IGF-1, microbiota–gut–brain, epigenetic, and intestinal stem-cell pathways remain largely supported by indirect evidence. Practical feeding decisions should match a characterized preparation to a defined objective and evaluate the intended benefit alongside performance, stability, and safety.

## Figures and Tables

**Figure 1:**
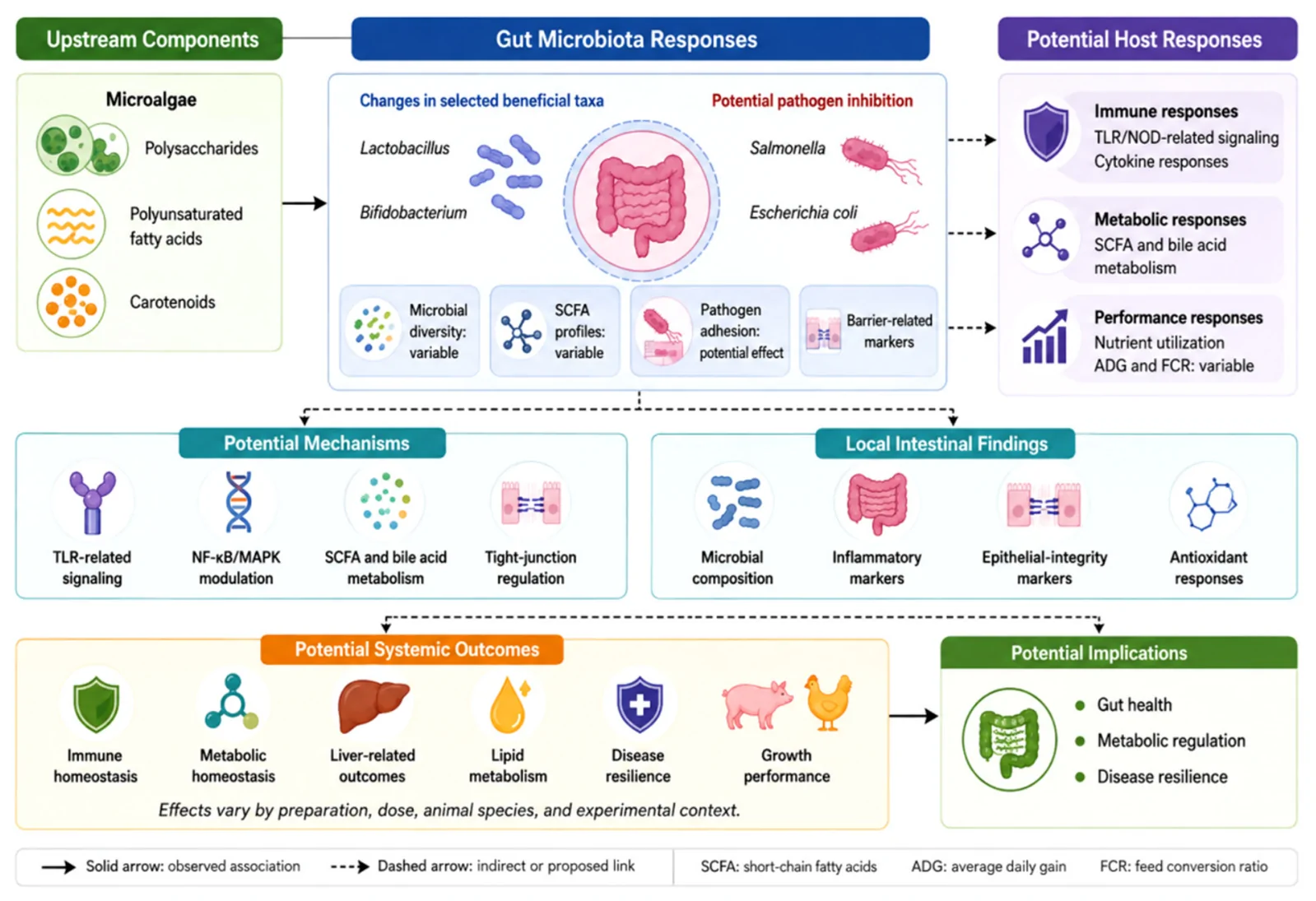
Direct and potential effects of algal bioactive compounds on the livestock gut microbiota. Changes in microbial composition and fermentation metabolites have been directly observed for selected algal preparations in specific animal models, whereas several downstream metabolic and host-response pathways remain indirect or proposed. Solid arrows indicate direct livestock or poultry evidence, dashed arrows indicate indirect evidence, and dotted arrows indicate proposed relationships.

**Figure 2:**
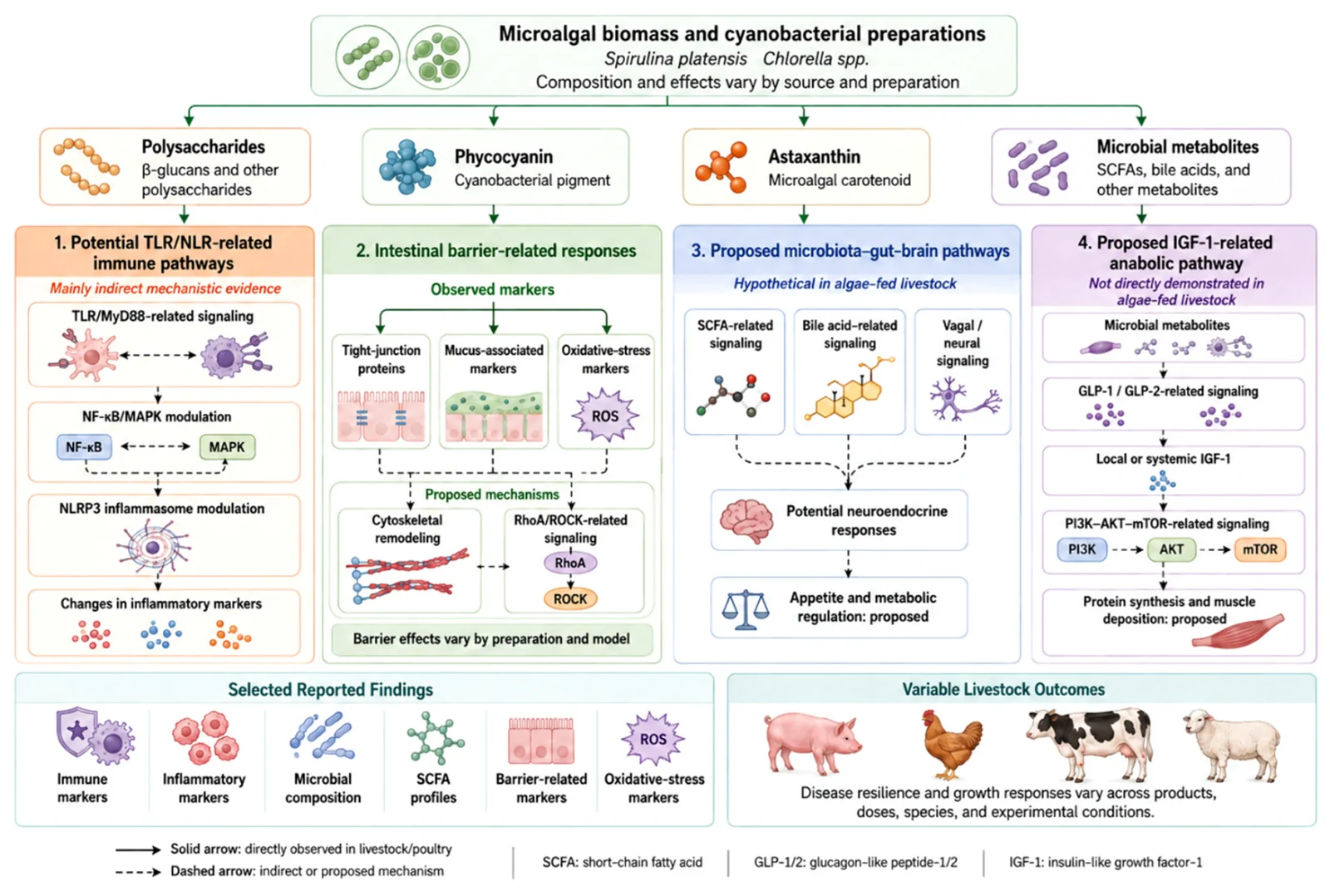
Direct, indirect, and proposed pathways potentially linking algal bioactive compounds to livestock gut health. Available livestock evidence supports associations between selected algal preparations and changes in intestinal barrier markers, immune responses, microbial composition, and animal performance. However, specific pathways involving TLR (Toll-like receptor)/NLR (NOD-like receptor) signaling, MyD88 (myeloid differentiation primary response 88), NF-κB (nuclear factor kappa B), MAPK (mitogen-activated protein kinase), SCFAs (short-chain fatty acids), GLP-1/2 (glucagon-like peptides 1 and 2), IGF-1 (insulin-like growth factor 1), PI3K (phosphoinositide 3-kinase), AKT (protein kinase B), mTOR (mechanistic target of rapamycin), ROS (reactive oxygen species), RhoA (Ras homolog family member A), and ROCK (Rho-associated coiled-coil-containing protein kinase) are supported to varying degrees by livestock, rodent, cell-culture, or indirect evidence. Solid arrows indicate direct livestock or poultry evidence, whereas dashed arrows indicate indirect or proposed mechanisms.

**Figure 3:**
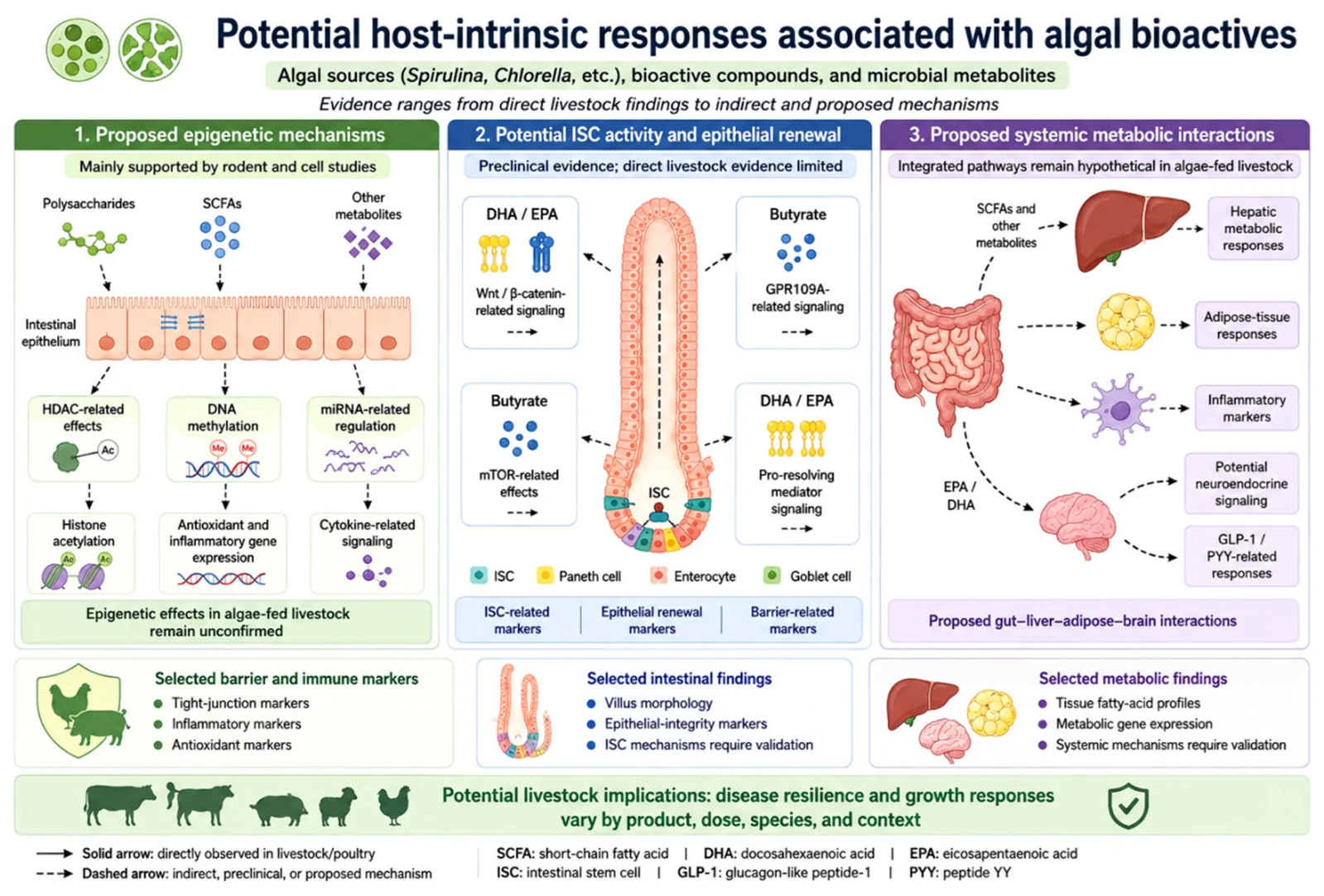
Proposed host-intrinsic molecular responses associated with algal bioactives and microbial metabolites. Epigenetic regulation, intestinal stem-cell activity, epithelial renewal, and gut–liver–adipose or microbiota–gut–brain signaling represent potential mechanisms. Most of these pathways are currently supported by rodent, cell-culture, organoid, isolated-compound, or other indirect evidence rather than by direct studies in algae-fed livestock. Solid arrows indicate direct livestock or poultry evidence, dashed arrows indicate indirect evidence, and dotted arrows indicate proposed relationships.

**Table 1:** Algal bioactive components, reported responses, and proposed mechanisms related to livestock gut health.

Algal Bioactive Components	Reported or Proposed Mechanisms	Gut Microbiota Modulation	Host Physiological Effects	References
Polysaccharides	Serve as microbiota-accessible carbohydrates; fermented into SCFAs	Promote Lactobacillus and Bifidobacterium; reshape microbial composition	Maintain microbial homeostasis and intestinal metabolic environment	[7,12,14]
β-glucans and sulfated polysaccharides	Structure-dependent regulation of microbial metabolism and immunity	Influence beneficial microbial taxa	Potential immunomodulatory effects	[11,13]
EPA/DHA (ω-3 PUFAs)	Regulate PPARγ and NF-κB pathways	Influence the microbial environment and pathogen inhibition	Protect tight-junction proteins and reduce inflammation	[17,18]
Carotenoids (astaxanthin)	Scavenge ROS and enhance antioxidant defense	Improve oxidative environment	Increase CLDN1 and MUC2 expression	[19,20]
Phycocyanin	Modulate oxidative stress and inflammatory pathways	Improve intestinal microenvironment	Increase antioxidant capacity	[21,28]
Whole algal biomass	Multi-component synergistic effects	Alter microbial composition and metabolism	Improve intestinal morphology and performance	[5,10]

**Table 2:** Application effects, molecular mechanisms, and challenges of algal biomass and algal-derived preparations in livestock production.

Animal Model/Application	Algal Biomass or Preparation	Observed Effects	Potential Mechanisms	Current Limitations	References
Broiler chickens	*Spirulina platensis*/*Chlorella vulgaris*	Changes in cecal microbiota; improved antioxidant status and intestinal barrier markers	Microbial fermentation regulation, antioxidant pathways, and immune modulation	Effects depend on dose, processing, and dietary background	[10,22]
Weaned piglets	*Chlorella vulgaris*/*Spirulina*	Altered bacterial composition and intestinal morphology; variable growth responses	Microbiota remodeling, nutrient utilization, and intestinal development	High inclusion levels may reduce digestibility and performance	[14,37,39]
Ruminants	DHA-rich microalgae and algal products	Effects on fatty acid utilization and rumen microbial composition	Modification of rumen microbial ecology and lipid metabolism	Biohydrogenation, rumen disturbance, and long-term safety require evaluation	[47,48]
Industrial production	Algal biomass and extracts	Potential sustainable feed ingredient	Optimization of cultivation, harvesting, drying, and stabilization technologies	High production cost, bioactive compound stability, and contamination control	[51,55]

Note: Mechanistic interpretations draw on evidence of varying directness. Findings from rodent, cell-culture, organoid, or non-algal intervention studies do not establish the same mechanism in livestock receiving algal preparations. Results obtained with isolated compounds should not be assumed to explain responses to whole biomass.

**Table 3:** Dose- and product-dependent responses to algal biomass and algal-derived preparations in livestock and poultry.

Animal Model	Algal Product and Dose	Main Observed Outcomes	Evidence Interpretation	Refs.
Broiler chickens	*Spirulina* (*Arthrospira platensis*); 0.25–1.0% of diet	Dose-dependent increase in cecal Lactobacillus; no direct demonstration of improved growth or pathogen exclusion	Microbiota response under the tested conditions; broader benefits remain uncertain	[22]
Weaned piglets	*Spirulina*; 1% of diet	Improved gross-energy digestibility and trends toward improved nutrient digestibility; no significant improvement in growth or diarrhea incidence	Mixed response; no demonstrated performance or antidiarrheal benefit	[35]
Piglets around weaning	*Spirulina*; oral administration at approximately 385 mg/kg body weight/day	Reduced occurrence of post-weaning diarrhea and increased body-weight gain during the suckling period	Favorable response under a specific oral-administration regimen	[36]
Post-weaning piglets	*Spirulina*; 10% of diet	Reduced final body weight and average daily gain; impaired feed conversion; increased digesta viscosity and reduced protein digestibility	Adverse response at the tested high inclusion level	[37,38,39]
Broiler chickens	*Spirulina*; 15% of diet	Reduced body-weight gain and impaired feed conversion, associated with increased digesta viscosity	Adverse response at the tested high inclusion level	[40,41]
Broiler chickens	DHA-rich microalgae; 2% of diet	Increased breast-muscle n-3 PUFA content; methionine co-supplementation reduced white striping and myopathic lesions without impairing growth	Product-quality effects; no demonstrated growth enhancement	[42]
Broiler chickens	*Aurantiochytrium acetophilum*; 4% of diet, providing 6.8 g DHA/kg diet	Reduced body-weight gain, feed efficiency, and breast-muscle weight; increased lipid oxidation	Adverse response at the tested DHA supply and inclusion level	[43]
Broiler chickens	*Chlorella vulgaris;* 10% of diet	Growth was not compromised; meat yellowness, carotenoid content, and tenderness increased; no consistent additional benefit from CAZyme supplementation	Product-quality effects without a consistent additional enzyme benefit	[44]
Laying hens	*Nannochloropsis limnetica*; 1–3% of diet	Dose-dependent increase in egg-yolk EPA and DHA; laying rate improved by 2–3% without adverse effects on egg quality or nutrient retention	Dose-dependent product-quality response under the tested conditions	[46]
Beef steers	*Asparagopsis taxiformis*; study-specific inclusion levels	Methane yield decreased, with the magnitude depending on inclusion level, diet composition, and bromoform concentration	Product- and context-specific methane response; long-term safety requires evaluation	[49,50]

Note: The table provides a structured narrative comparison rather than a formal quantitative dose–response analysis. Responses are specific to the tested product, dose, administration route, animal model, diet, and experimental conditions. CAZyme, carbohydrate-active enzyme; DHA, docosahexaenoic acid; EPA, eicosapentaenoic acid; PUFA, polyunsaturated fatty acid.

## Data Availability

No new data were created or analyzed in this study. All information discussed in this review article is derived from publicly available published literature cited within the manuscript.
